# DeleteROI for Cleaning CiliaQ Output of Non-ciliary Contamination

**DOI:** 10.17912/micropub.biology.001670

**Published:** 2025-08-14

**Authors:** Jeffrey J. Anuszczyk, Michael W. Stuck, Thibaut Eguether, Gregory J. Pazour

**Affiliations:** 1 Program in Molecular Medicine, University of Massachusetts Chan Medical School, Worcester, Massachusetts, United States; 2 Inserm UMS55 ART ARNm, Inserm UMS55 ART ARNm, LI2RSO, Université d’Orléans, CHU d’Orléans, 45100 Orléans, France

## Abstract

The ImageJ plugin CiliaQ developed by Hansen and colleagues (Hansen et al., 2021) provides for sophisticated analysis of ciliary parameters in three-dimensional space. However, midbodies and other non-ciliary structures can contaminate the output and require significant effort to remove. Furthermore, the manual removal of contamination risks subjective bias as the data is not blinded to the investigator. To address these problems, we developed an ImageJ plugin that presents images of the cilia region-of-interests (ROIs) identified by CiliaQ in a clickable grid that allows for marking and automated removal of non-ciliary contaminants. To reduce subjective bias, our plugin works on a dataset of multiple images and presents the cilia ROIs randomly. If the dataset contains both control and experimental conditions, the cilia are randomly interspersed with no visible information about their experimental group, thus reducing subjective bias. After removal of contamination, the cleaned data is output maintaining the CiliaQ file formats initially used.

**Figure 1. DeleteROI Montage:**
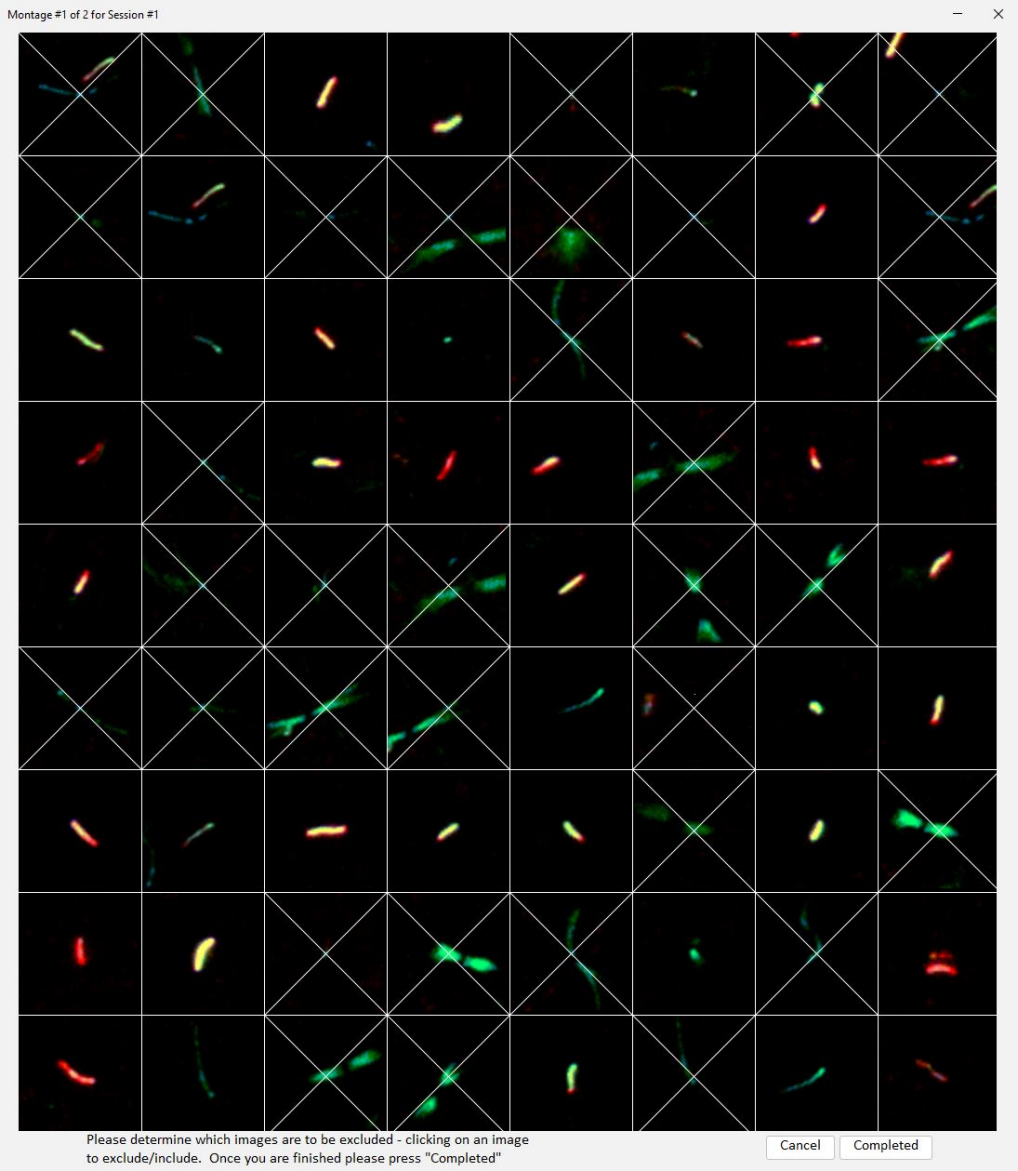
DeleteROI presents the cilia ROIs in a montage. Clicking on ROIs in the montage marks them for removal from the dataset. A second click will restore them to the dataset. **Cancel** will terminate the process and exit DeleteROI. Once all ROIs have been examined, Selecting **Completed** will move to the next montage in the group. Text at the top lists the number of montages in this group and the number of groups in the dataset.

## Description

**Installation and Operation**

CiliaQ-DeleteROI is intended to be used after the third CiliaQ module, which is simply called CiliaQ. The user is expected to have used the CiliaQ Preparator or an equivalent method to segment cilia in a series of images and processed them through CiliaQ. The use of the CiliaQ Editor is optional and may be needed to improve segmentation. There is little value to remove segmented objects with the CiliaQ Editor as this is handled more efficiently with CiliaQ-DeleteROI.

CiliaQ-DeleteROI is dependent on FIJI (Schindelin et al., 2012) and the output from CiliaQ (Hansen et al., 2021). After downloading the DeleteROI_.jar file from GitHub, use the FIJI **Plugins->Install...** (not **Install Plugin...** ) to install this module. Windows users will need to be running FIJI in administrator mode for this operation.

Restart FIJI and run the software via **Plugins -> DeleteROI** . A popup menu will allow you to navigate to a folder containing your images and the CiliaQ output files. DeleteROI uses two files. The first is a tif image that ends with CQ_RP.tif or CQ_date_time_RP.tif containing the ciliary masks and the immunofluorescence channels. The second is a spreadsheet generated by CiliaQ that ends with CQ.txt or CQ_date_time.txt. Note that the txt file can be in either US (0.00…) or German (0,00…) format and this will be maintained. The menu allows three options to control how the upcoming grid of ROIs is displayed. The first option “Saturation” controls the percentage of the pixels that are saturated with higher numbers producing more saturated images. The second “Min/Max” sets minimal and maximal display values for each of the three channels. The third “Manual” allows you to view the tif image and adjust on an image-by-image basis.

Once you have selected the folder containing your images and chose the display settings, use **Update** to start the process. DeleteROI will survey your folder and will update the popup to present the number of images and ROIs to be processed from each of the tifs in the folder. Select **Next** to continue. If there are more than 500 ROIs, the data will be presented in sessions. At the end of each session, the analysis is stored so if there is an interruption, the completed sessions do not have to be reanalyzed. Advanced Options allow for scaling the ROIs and determining how big the ROI needs to be. 32 pixels and 3x are good options for 40x images of fibroblast cilia. Higher magnification or longer cilia would require larger ROIs and possibly smaller scales. Advanced options also allow one to enter Debug mode where the tif images remain open and the ROIs will have an identifier included to track them back to the originating tif image. This allows one to see the ROI in context of the image, which can be useful in initial efforts to understand what ROIs represent cilia but removes the blinding of the data. By default, the image name is stored in column 1 of the CQs file but this option can be changed on the advanced option screen.

Once **Next** is selected, a montage grid of ROIs will be presented (Fig 1). These ROIs are randomly chosen from the dataset to reduce subjective bias. Clicking on ROIs of contamination will put an X over them and will mark them for removal from the dataset. Clicking a second time will remove the X and allow them to remain in the dataset. Select **Completed** to move to the next group of ROIs and continue until the session is completed. Once the session of about 500 ROIs is completed, a new set of files will be written to a subfolder called “Group_1” that will contain two new spreadsheets. One, ending CQ.txt, will be similar to the original CQ.txt CiliaQ file except that the ROIs that were selected for removal are marked with an # in column 1 effectively commenting them out. This file is duplicated in the original folder as CQ-active.txt. The second, CQ-stripped.txt, only contains the active entries from CQs.txt CiliaQ file plus the addition of the image name in column 1 (or wherever was selected in the advanced options). The CQ-stripped.txt file is intended to be directly used for secondary data processing. The original CQ.txt file remains unchanged in the original folder. A text file (README.txt) with the metadata of the session and deletion history is also present in the folder.

The design of DeleteROI allows for multiple passes through the data to cull additional ROIs without having to start over. A second pass can be useful when the first pass was too conservative, and more ROIs need to be removed. Alternatively, it can be used for subsequent subclassification of cilia with a particular property e.g. positive for an experimental marker. Each pass through the data creates a subfolder “Group_x” with x incremented with each pass and the CQ-active file updated with the most recent iteration. To run subsequent passes through the data, run DeleteROI as before, pointing to the same folder or images, and select **New Session** after selecting **Update** .

**Code Availability**

Code is available at https://github.com/pazourg/DeleteROI . A practice dataset of cilia labeled with acetylated tubulin and Arl13b is available at https://Cilia.Pro/DeleteROI/ExampleData . More detailed descriptions of the popups is described in https://github.com/pazourg/DeleteROI/tree/main/Documentation/ .

**Conflict of Interest**

The authors have no conflicts of interest in this work.

## Data Availability

Description: Example datasets for use with the DeleteROI plugin for ImageJ/FIJI. This data includes '.tif' image files and corresponding '.txt' metadata files that demonstrate how the DeleteROI plugin works with real-world input.. Resource Type: Dataset. DOI: https://doi.org/10.22002/gkpc0-sk256 Description: Plugin Code. Resource Type: Software. DOI: https://doi.org/10.22002/c9j15-r7v75
