# Erratum: DeleteROI for Cleaning CiliaQ Output of Non-ciliary Contamination

**DOI:** 10.17912/micropub.biology.001839

**Published:** 2025-09-04

## Description

The Extended Data file, DeleteROI-v1.0.3-2.zip, containing the DeleteROI plugin code, was mistakenly not appended to the original publication. We have now included that file with the article. 

